# Inertial Focusing of Microparticles in Curvilinear Microchannels

**DOI:** 10.1038/srep38809

**Published:** 2016-12-19

**Authors:** Arzu Özbey, Mehrdad Karimzadehkhouei, Sarp Akgönül, Devrim Gozuacik, Ali Koşar

**Affiliations:** 1 Faculty of Engineering and Natural Science, Molecular Genetics and Bioengineering Program, Sabanci University, Orhanli-Tuzla, Istanbul, 34956, Turkey; 2 Faculty of Engineering and Natural Science, Biological Sciences and Bioengineering Program, Sabanci University, Orhanli-Tuzla, Istanbul, 34956, Turkey; 3 Center of Excellence for Functional Surfaces and Interfaces for Nano-Diagnostics (EFSUN), Sabanci University, Orhanli-Tuzla, Istanbul, 34956, Turkey

## Abstract

A passive, continuous and size-dependent focusing technique enabled by “inertial microfluidics”, which takes advantage of hydrodynamic forces, is implemented in this study to focus microparticles. The objective is to analyse the decoupling effects of inertial forces and Dean drag forces on microparticles of different sizes in curvilinear microchannels with inner radius of 800 μm and curvature angle of 280°, which have not been considered in the literature related to inertial microfluidics. This fundamental approach gives insight into the underlying physics of particle dynamics and offers continuous, high-throughput, label-free and parallelizable size-based particle separation. Our design allows the same footprint to be occupied as straight channels, which makes parallelization possible with optical detection integration. This feature is also useful for ultrahigh-throughput applications such as flow cytometers with the advantages of reduced cost and size. The focusing behaviour of 20, 15 and 10 μm fluorescent polystyrene microparticles was examined for different channel Reynolds numbers. Lateral and vertical particle migrations and the equilibrium positions of these particles were investigated in detail, which may lead to the design of novel microfluidic devices with high efficiency and high throughput for particle separation, rapid detection and diagnosis of circulating tumour cells with reduced cost.

Microfluidic technologies have many advantages over conventional technologies such as the requirement for a small sample volume, low-cost production, higher sensitivity and improved performance^1^,^2^. They take advantage of the fact that the flow characteristics at the microscale may be appealingly different from those at the macroscale such that the dominant forces in microfluidics may become negligible at the macroscale^3^. Furthermore, microfluidic technologies facilitate the fabrication of integrative, portable point-of-care (POC) diagnostic devices based on lab-on-a-chip or micro-total-analysis-systems (μTAS)^4^. These devices contribute great benefit to biomedical research in the detection, sorting, separation and analysis of cells, especially circulating tumour cells (CTCs) to provide effective diagnosis and therapy^5^,^6^,^7^.

Circulating tumour cells (CTCs) are rare cancer cells which originate from the primary tumours and interfered to bloodstream. Isolation of CTCs from blood is critical owing to the fact that metastatic CTCs may hold different genomic and phenotypic properties which may provide insights for prognosis and effective treatment. Focusing biological particles and cells using microfluidic systems have been implemented as an efficient CTCs enumeration and enrichment method for clinical diagnostics applications^8^,^9^. Focusing particles and cells into a narrow stream is a requirement for these emerging applications and for understanding the underlying physics of particle/cell focusing in microfluidics^10^.

A variety of fundamental focusing and separation approaches have been studied with synthetic microparticles in the framework of microsystems^11^,^12^,^13^. From the microfluidics point of view, separation/isolation principles are divided into two categories depending on the external energy usage: active and passive separation^13^. Whereas active techniques require external forces such as magnetic^14^,^15^, dielectric^16^,^17^ and acoustic^18^,^19^ forces to separate particles/cells, passive techniques mainly utilize hydrodynamic forces^20^. Passive techniques can be further separated into filtration-based, deterministic lateral-displacement-based and inertia-based techniques^6^. Noticeably, active techniques provide more accurate results yet are limited by their low throughput, integration of complex components and expensive production or process requirements^21^. Several recent reviews on microfluidics particle/cell focusing and isolation have enhanced our understanding of separation characteristics and physics^5^,^10^,^11^,^12^,^13^,^21^. Among these techniques, inertial focusing has gained significant attention because it offers high throughput and effective and precise control for particle and cell manipulation. Despite being an actively studied topic, inertial particle focusing behaviour and its underlying mechanisms are not yet fully understood. Different channel types, such as straight^22^,^23^,^24^,^25^,^26^, serpentine^27^,^28^,^29^,^30^,^31^,^32^,^33^, spiral^34^,^35^,^36^,^37^,^38^,^39^,^40^,^41^ and straight with contraction–expansion arrays^42^,^43^,^44^,^45^,^46^,^47^, are used in inertial microfluidics, however the separation of particles with a serpentine microchannel has not attracted as much attention as the other types. In serpentine channels, secondary flow directions vary with a sudden change in the channel curvature. As a result, steady state secondary flows cannot be precisely assessed. Recently, the highest efficiency was found as 95% by the Nguyen’s group^48^. However, the throughput was not as much as that in spiral channels^25^,^38^,^39^.

The Dean drag force is introduced by using a curvilinear channel geometry. The effect of this curvilinear geometry emerges with the formation of two counter-rotating vortices, Dean vortices, which exert a drag force on the particles. This force is directed outwards near the channel centre and inwards near the upper and lower walls^41^,^49^. The radial circulation of these Dean vortices is directed towards the outer wall at the midline, while it is directed towards the inner wall at the top and bottom regions of the channel.

In contrast to the studies on inertial microfluidics in the literature, the effect of curvilinearity with a high curvature angle (280°) on particle focusing behaviour is examined in this study by performing inertial focusing of 10 μm, 15 μm and 20 μm fluorescent polystyrene microparticles at different channel Reynolds numbers. Furthermore, the decoupling effect of inertial and Dean drag forces on particles and separation potential are revealed. Because the forces acting on the particles vary depending on their location, the concomitant effect remains unknown. This study has the potential to provide a valuable contribution to the field of inertial microfluidics by extensively improving our understanding of three-dimensional particle dynamics in curvilinear channels. We have developed a continuous, high-throughput and parallelizable size-based particle focusing technique with high separation potential in a specific symmetrical curved channel by taking advantage of inertial microfluidics and Dean flow physics. Our design allows almost the same footprint to be occupied as straight channels, which enables parallelization with parallel optical detection integration and may lead to the design of novel microfluidic devices with high efficiency and high throughput similar to spiral channels for particle separation.

## Working Principle

Since the discovery of the tubular pinch effect^50^,^51^, many studies have explained this behaviour in the literature^52^,^53^,^54^,^55^. Accordingly, equilibrium positions arise from a balance between the shear gradient lift force and the wall-induced lift force. Wall-induced and shear gradient lift forces, which act on a particle and are orthogonal to the flow directions, depend on the particle position and the channel Reynolds number. The particles in a straight fluid flow experience an external force called inertial lift force. This inertial lift force can be divided into two forces: shear gradient lift force *F*_*S*_ and wall-induced lift force *F*_*W*_. The shear gradient inertial lift force occurs owing to the parabolic velocity profile of the resulting Poiseuille flow, which leads to the migration of particles/cells from the microchannel centre towards the microchannel walls. In contrast, the wall-induced lift force acts in the opposite direction, fading towards the microchannel centre. For successful inertial focusing, previous studies reported a minimum threshold of *λ* = *a*_*p*_/*D*_*h*_ > 0.07 and 
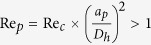
, where *a*_*p*_ is the diameter of the particle, *D*_*h*_ is the hydraulic diameter of the channel, Re_*p*_ and Re_*C*_ are the Reynolds numbers of the particle and channel, respectively^33^.

In Poiseuille flows, the net lift force acting on the particle is given by


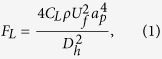


where 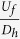 is the shear rate and *C*_*L*_ is the lift coefficient, which is a function of the Re_*C*_ and changes in the range 0.2–0.5 in microfluidic applications^38^,^55^. Recently, it was found that the lift force scaling is bound to the particle position in the channel such that the shear gradient force dominates in the movement of the particles near the centreline of the channel^33^. The net lift force scaling is expressed near the channel centreline as


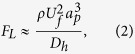


where as it becomes


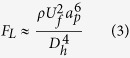


near the channel walls^56^.

The equilibrium positions can be further controlled using curved channels^57^,^58^. A dimensionless number called the Dean number (*De*) is used to quantify the magnitude of these two vortices and is given by


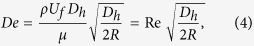


where *μ* is the fluid viscosity, *U*_*f*_ is the mean fluid velocity, *R* is the curve radius of the channel, and Re_*C*_ is the dimensionless channel Reynolds number. Combining the magnitudes of the net lift force and the Dean drag force reduces the number of equilibrium positions to one with a controlled Re_*C*_. Recent comprehensive reviews about the underlying physics of particle dynamics in inertial microfluidics can be found elsewhere^23^,^49^,^57^,^58^,^59^.

## Results

**Figure 1:**
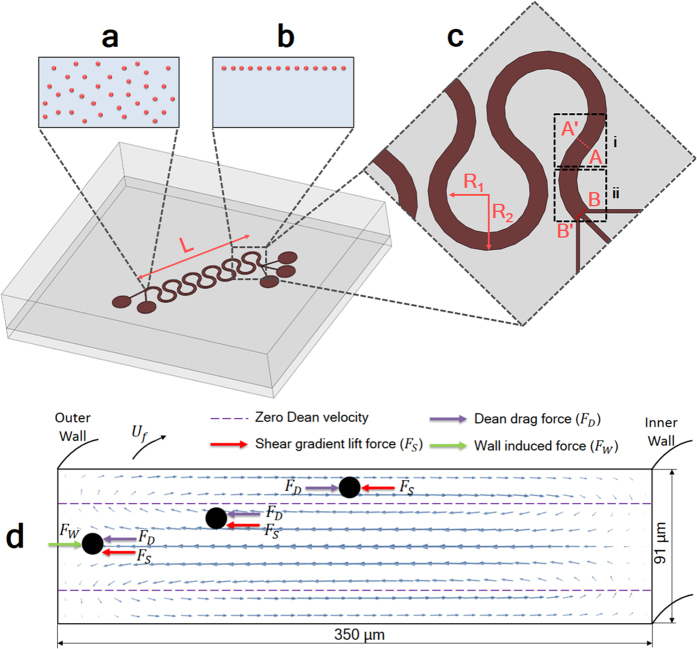
Schematic of the particle focusing device. The randomly dispensed particles in the inlet region (**a**) focus into a single particle stream at the AA’ cross section (at the transition region) (**b**). (**c**) A close-up view of the channel transition region (i) and the outlet region (ii). (R_1_ = 800 μm and R_2_ = 1150 μm and L = 9.75 mm) (**d**) A force diagram depicting the direction of forces acting on the particle of various locations.

The PDMS microfluidics chip used in this study consists of one microchannel design that includes two inlets and three outlets with diameters of 1 mm each. The height and width of the microchannel are 91 μm and 350 μm, respectively. The whole chip comprises 11 curvilinear geometries, each having a curvature inner radius of 800 μm and a curvature angle of 280°. The total chip length is approximately 4.3 cm. A schematic of the chip is shown in Fig. 1.

Our experimental observations demonstrate the need for the analysis to be focused on two specific channel regions: the transition region, Fig. 1c(i), and the outlet region, Fig. 1c(ii). Owing to the sudden rotational change in the transition region, the outer wall becomes the inner wall and vice versa. Thus, to prevent confusion, as illustrated in Fig. 1c, it is best to define the wall between A and B as wall W_1_ and the wall between A’ and B’ as wall W_2_.

**Figure 2:**
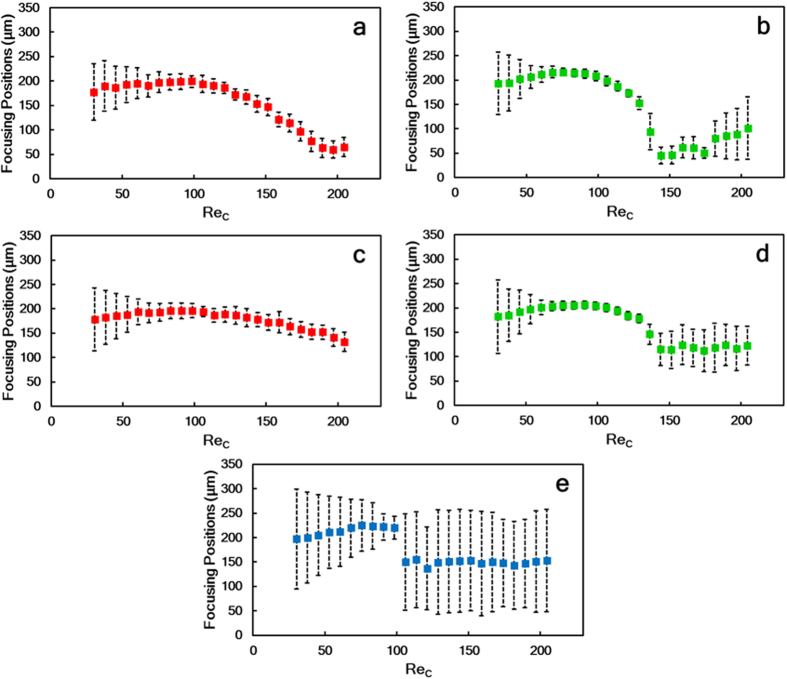
Focusing positions of microparticles of three different sizes for the curved microchannel with varying flow rates and corresponding channel Reynolds numbers in the two aforementioned regions: the outlet region for particles of sizes (**a**) 20 μm (red), (**b**) 15 μm (green), (**c**) 10 μm (blue) and the transition region for (**d**) 20 μm (red) and (**e**) 15 μm (green) particles. Plotted error bars indicate the width of the focusing streak.

To investigate the effects of curvature on microparticle focusing of microparticles of different sizes, sets of experiments were performed for three different sizes (20 μm (large), 15 μm (medium) and 10 μm (small)) at a wide range of flow rates varying between 400 μL/min and 2700 μl/min and corresponding to Reynolds numbers between 30 and 205. Figure 2 presents the particle streak positions and focusing width data in the outlet and transition regions. In the figures related to both the transition and outlet regions, the bottom and top of each figure represent walls W_2_ and W_1_, respectively.

Initially, at the lowest Re_*C*_ (Re_*C*_ = 30), both large (*a*_*p*_ = 20 μm; *λ* = 0.139) and medium (*a*_*p*_ = 15 μm; *λ* = 0.1) particles pass along the channel by occupying the centre of the channel as a wide band, and have a focusing width more than four times the diameter of a single particle. With an increase in Re_*C*_ (to 76) for large particles and (to 60) for medium particles, the focusing width decreases to a single streak with a thickness of approximately less than twice the diameter of a single particle. No considerable change is observed in the focusing width of large particles for both transition and outlet regions with a further increase up to the highest value (Re_*C*_ = 205), (Fig. 2a,c). Medium particles start defocusing in the outlet region, when Re_*C*_ > 136 (Fig. 2d). Because of the bypassing movement of medium particles, more sophisticated results can be seen in Fig. 2b in the transition region by increasing the Re_*C*_ numbers. A sudden increase in the focusing width is seen at Re_*C*_ = 136, while a refocusing trend is observed with a focusing width of almost twice the diameter of a single particle (at Re_*C*_ = 144). With a further increase in Re_*C*_ (Re_*C*_ > 182), focusing is disturbed by the dominance of the Dean drag force.

The focusing width of small particles (*a*_*p*_ = 10 μm; *λ* = 0.07) is observed to be in uniform alignment throughout the microchannel at low Reynolds numbers and tends to decrease in size as the Re_*C*_increases to 98. At this Re_*C*_ number, the focusing width is almost four times greater than the diameter of a single particle. However, a further increase in the Re_*C*_ results in a defocusing trend of the small particles in the outlet region.

Notwithstanding that the focusing position of large particles is very close to the centreline of the microchannel for all Re_*C*_ at the outer region, this trend is disturbed, and the particle stream approaches W_2_ at the transition region when the Re_*C*_ is greater than 98. Care must be taken when interpreting these results because the large particles are observed to be in the equilibrium position near the centreline, owing to the directional magnitude balance between the shear gradient lift and Dean drag forces; however, when the Re_*C*_ > 98, particles tend to pass through the outer wall upstream and downstream of the transition region. Thus, the particles approach the centreline at the outlet, where the data are taken (B-B’ cross section).

The tightly focused stream of the medium particles initially forms close to the centreline, slightly on the W_1_ side of the channel in both the transition and outlet regions, when the Re_*C*_ is 61. Increasing the Re_*C*_ causes the particle equilibrium position to shift through the W_2_ side up to Re_*C*_ ~ 136. However, at this Reynolds number (Re_*C*_ ~ 136), a sudden increase in the focusing width is observed in both regions between the centreline and the W_2_ region. Although this defocusing observation continues up to the highest Re_*C*_ in the outlet region, interestingly, a tightly focused streamline is regenerated near the W_2_ for a Re_*C*_ of 144 in the transition region.

**Figure 3:**
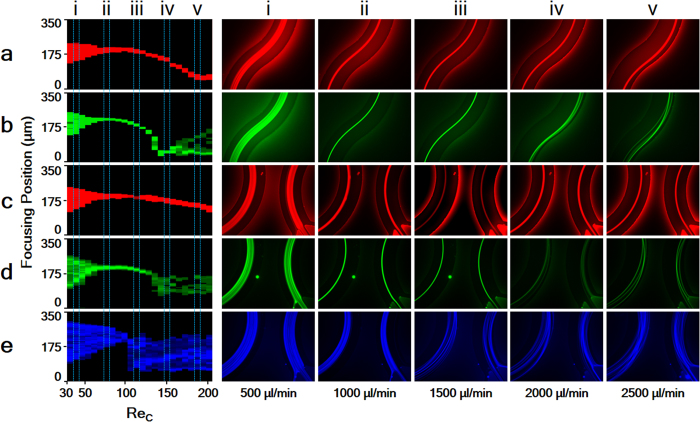
Re_*C*_ maps: at the transition region for (**a**) 20 μm (red) and (**b**) 15 μm (green) particles and at the outlet region (**c**) 20 μm (red), (**d**) 15 μm (green) and (**e**) 10 μm (blue) particles. Particle streams for different channel Reynolds numbers corresponding to five selected flow rates between 500 and 2500 μL/min (i–v).

At the lowest Reynolds number, the focusing width of small particles is greater than that of large and medium particles. With an increase in Re_*C*_ number to 98, a decreasing trend can be seen in the focusing width. A sudden defocusing behaviour is observed with a further increase in Re_*C*_, (Fig. 3e). Prior to this defocusing behaviour, the wide particle streak starts to approach wall W_1_ with an increasing Re_*C*_. However, this streak is then disrupted by the wall W_2_, which suggests that there is a rotation to the opposite direction of one of the dominant forces, thereby balancing the other dominant forces.

The Re_*C*_ maps and representative row images provided in Fig. 3 present a better understanding of particle focusing behaviour over a wide range of channel Reynolds numbers. These maps are obtained by taking the intensity line graphs, where the line is on the A-A’ cross-section for the transition region and on the B-B’ cross-section for the outlet region at different flow rates. Following that, the counter graph, which gives pixel maps corresponding to the intensity line graph data, is obtained. Re_*C*_ maps for both the transition and outlet regions are constructed frame by frame with the bottom and top edges of each graph representing walls W_2_ and W_1_, respectively.

Large particles initially form a wide particle streak band near the centreline of the channel with a low Re_*C*_ (Re_*C*_ ~ 38), Fig. 3a(i),c(i). At this stage, owing to low flow velocity, the particle motion behaviour is slightly influenced by the Dean drag and inertial lift forces. Increasing the Re_*C*_ (Re_*C*_ ~ 75) results in the formation of a single stable particle stream near the centreline (falling on the W_1_ side) along the microchannels. Owing to an increase in the shear gradient lift force, this stream migrates to wall W_2_ in the transition region. After a further increase in the Re_*C*,_ a transverse motion of focusing particles is observed. Particles moving into the transition region do not migrate along the essential flow stream but instead migrate perpendicular to this stream as they move closer to the outer wall rather than remaining at the channel centreline. Although this position is preserved at the transition region, and the particle stream remains close to W_2_, the Dean vortices directions change with the sudden switch of the channel curvature, and, thus, the particle location is disturbed as they move to the centreline from W_2_ in the outlet region. The higher the flow rate becomes, the closer the particles move to W_2_ in the transition region.

Similar to large particles, medium particles are initially focused near the centreline at a low Re_*C*_ and form an equilibrium line that is closer to W_1_ than that of large particles. However, this streak does not migrate as it does for large particles. Instead, sudden defocusing behaviour is observed at Re_*C*_ ~  136; see Fig. 3b. Following this transition behaviour, particles are refocused near W_2_ at Re_*C*_ ~ 144. When the Re_*C*_ is further increased, a second defocusing effect can be observed in Fig. 3b(v) and d(v) owing to the dominant Dean drag force.

During all experiments, no effective focusing line was observed for small particles. However, the bypassing movement was more dominant. This expected result agrees with the previous studies mentioned above, which report a minimum threshold of *λ* > 0.07 for inertial focusing behaviour to occur^29^. Even so, achieving a focusing width of almost four times the diameter of a single particle using small particles suggests the possibility of focusing small particles with precise geometries.

## Separation Potential

By looking at the streak positions of particles in the transition region (Fig. 2d and e), it can be clearly observed that the behaviour of particles is different from that in the outlet region. As illustrated in Fig. 2d, the focusing width of large (20 μm) particles decreases with increasing flow rate, down to approximately double the particle diameter. At later stages, the equilibrium line starts to change its position from the centre towards W_1_. The migration of medium (15 μm) particles to W_1_ is faster than that of the 20 μm particles with increasing flow rate in the transition region, so there is a considerable distance between the equilibrium lines of the large and medium particles at an optimal Reynolds number. However, this distance decreases at that optimum Reynolds number in the outlet region.

One of the main reasons of this distance in the transition region is vertical position difference of medium and large particles at a given Re_*C*_. When Re_*C*_ is low, the vertical positions of both large and medium particles are supposed to be above the zero velocity line, where the Dean drag force direction is from the outer wall to the inner wall and the shear gradient lift force balances the Dean drag force near the centreline. However, an increase in Re_*C*_ results in crossing medium particles the zero Dean velocity line. Beyond this vertical position, medium particles experience a switch in the direction of the Dean drag force, which is now directed towards the outer wall. Therefore, as particles approach to the transition region, the shear gradient force and Dean drag force act on medium particles in the same direction and cause a lateral migration through the W_2_, while they act on large particles in such a way that they balance each other near the centreline. In the outlet region, while the Dean drag force acts on large particles from the outer wall to the inner wall and balances the shear gradient lift force near the centreline, it acts on medium particles from the inner wall to the outer wall. The Dean drag force and the shear gradient lift force act on medium particles in the same way and cause a defocusing trend near the outlet region. A more detailed analysis can be found in the Discussion section.

**Figure 4:**
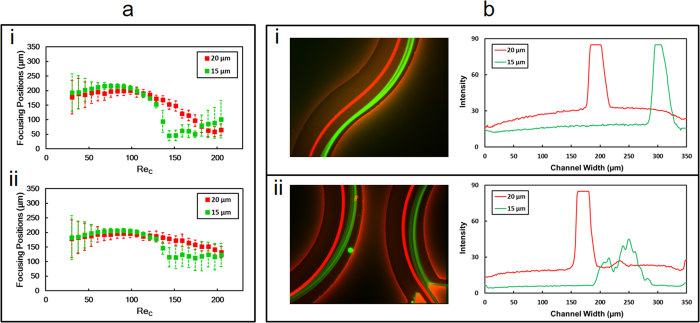
(**a**) Particle lateral position data from both 15 μm and 20 μm particles in the transition region (i) and in the outlet region (ii). (**b**) particle streams of 20 μm (red) and 15 μm (green) particles in the channel transition region (i) and in the outlet region (ii) when Re_*C*_ is 144; the corresponding intensity line graphs are presented to the right of each panel.

This study is a fundamental particle focusing study for better assessing the underlying physics of inertial microfluidics, which may lead to design novel microfluidic devices with a high separation efficiency. In such a curvilinear geometry, our findings suggest that designing the outlets of the curvilinear channel at the transition region could easily offer a better separation efficiency of particles with sizes of 20 μm and 15 μm. The optimal channel Reynolds number for such a separation of 20 μm and 15 μm particles is approximately 144 (Fig. 4) in the transition region. Furthermore, for this Reynolds number, the corresponding distance between the particle focusing streams of the large and medium particles is nearly 108 μm, which is three times the diameters of the particles combined together.

## Discussion

**Figure 5:**
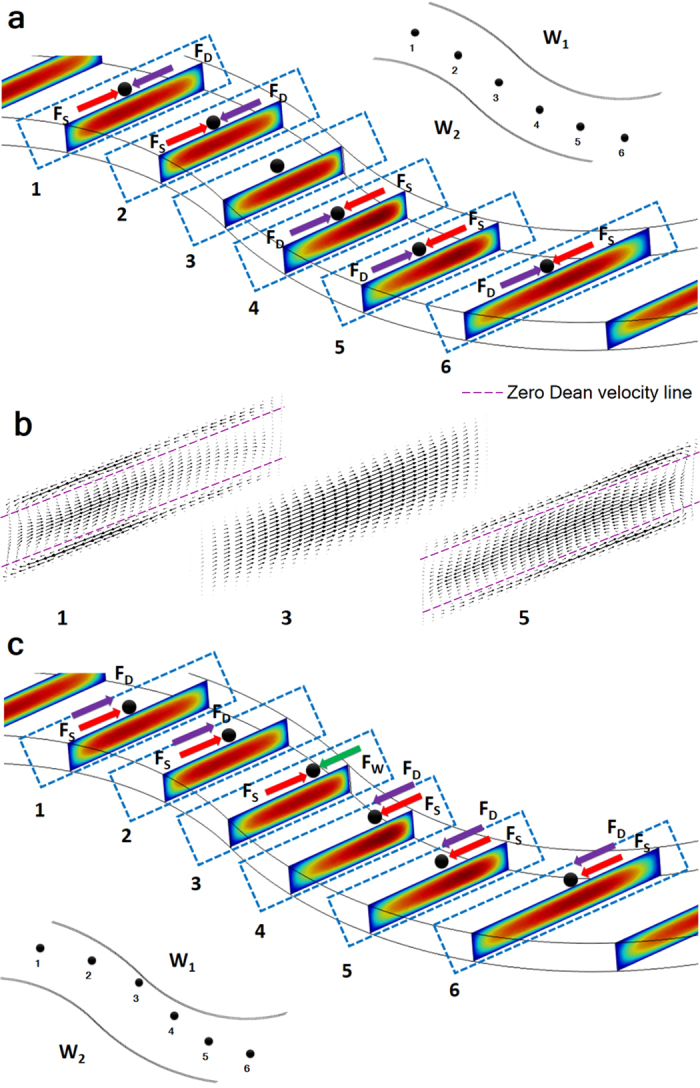
(**a**) Simulated velocity profile in the transition region and the forces acting on the particles where focusing occurs near the centreline. (**b**) Proportional Dean flow vectors with zero Dean velocity lines for particle positions 1, 3 and 5. (**c**) Simulated velocity profile in the transition region and the forces acting on the medium particles where transverse motion is observed. Dominant forces acting on the particles are *F*_*S*_ (shear gradient lift force), *F*_*D*_ (Dean drag force) and *F*_*W*_ (wall-induced lift force).

One of the main implications of this study is to establish the cruciality of the velocity profile shift with each turn and the effect of this change on the 3D position of the particle and the direction and magnitude of dominant forces acting on the particles. Previous simulation studies have shown that Dean vortices preserve a reasonably constant pattern and direction throughout the applied flow rates in spiral channels^39^. However, because of sudden turns in this case, the direction changes to the opposite direction rather than following a constant pattern. From a cross-sectional perspective, these two counter-rotating vortices follow a pathway that is parallel to the z-axis from the inner wall to the outer wall at the mid-section of the channel, whereas this pathway is in the opposite direction near the top and bottom walls as observed from planes 1 and 5 in Fig. 5b. The vertical position of the particles is predominantly influenced by the vertical component of the inertial lift force.

For the majority of the spiral geometry studies, the stable equilibrium line is observed close to inner wall^40^. Accordingly, the Dean drag force acts in the same direction as the net lift force near the outer wall, thereby causing particles to continue migrating along the circulation route of the two symmetric counter-rotating vortices. However, near the inner wall, the Dean drag force acts in the opposite direction, resulting in one stable equilibrium position of particles inside the microchannel. It should be noted that for this case, the particle’s vertical position must be close to the channel’s vertical centre because the direction of the Dean vortices is towards the channel outer wall near the channel’s vertical centre. In addition, the findings of Martin *et al*. on the effect of curvature ratios suggest a decrease in the shear gradient near the centreline as the velocity profile shifts due to an increase in curvature^41^. Because of this decrease, the particle’s vertical position moves closer to the centre on the inner half of the microchannel, resulting in the formation of an equilibrium position near the inner wall in spiral channels. Nevertheless, the case becomes more sophisticated for alternating curvilinear geometry because secondary flow directions vary with the sudden change in the channel curvature. Therefore, emerging steady state secondary flow conditions are not predictable precisely^58^,^59^. In straight channels, owing to the parabolic velocity profile, velocity maxima are formed at the centreline so that the magnitude of the lift coefficient and hence the lift force are zero at that point. However, the introduction of curvature to microchannels not only generates Dean drag to the force balance but also alters the velocity maxima. A single particle path is demonstrated in Fig. 5. When the particle is at position 1, velocity maxima are formed near the inner half of the channel. Towards to the transition region (position 3), velocity maxima shift through the centreline of the channel. In the transition region, the inner wall becomes the outer wall. Beyond this point, velocity maxima shift from the centreline through the inner wall as particle approaches to the outlet region (position 5). A continuous velocity maxima shift along the cross-section results in a differential change not only in the horizontal component but also in the vertical component of the shear gradient lift force, which continuously alters the 3D position of the particle. This effect is amplifying with a decrease in the particle size. It is well known that shear gradient lift force direction is from the velocity maxima through the channel walls.

The formation of single stable particle streams of both large and medium particles is observed near the centreline at different Re_*C*_. An increase in Re_*C*_, results in a transverse motion of large and medium particles. However, the force balance of large particles is different than that of medium particles during transverse motion. Although large particles remain focused until the maximum Re_*C*_, a defocusing trend is observed for medium particles when Re_*C*_ > 136. According to our results, there are three different cases which need to be discussed: (1) When both large and medium particle streams are near the centreline at lower Re_*C*_ (Fig. 5a), (2) transverse motion of medium particles at higher Re_*C*_ (Fig. 5c) and (3) transverse motion of large particles at higher Re_*C*_ (Fig. S2). For analysing the particle dynamics of these situations from a different perspective, force diagrams of all cases are prepared by using our simulation results. Force diagrams of cases 1 and 2 are preferred to be given in Fig. 5 while the force diagram of case 3 is presented in Fig. S2 because of its similarity to case 1.

In case 1 (Fig. 5a), when the particles are in position 1, where the velocity maxima are in the inner half of the channel, the horizontal component of shear gradient lift force pushes particles from velocity maxima to the outer wall. At this position, the particles’ vertical position is supposed to be above the zero Dean velocity line in the upper half of the channel and below the zero Dean velocity line in the lower half of the channel, where the Dean drag force direction is through the inner wall. Thus, the particles’ lateral position is observed near the channel centreline, where the shear gradient lift force and Dean drag force balance each other.

When particles move to position 2, the velocity maxima approach the channel centre, leading to a slight increase in the shear gradient lift force on the particles not only in the horizontal direction but also in the vertical direction. When the vertical component of the shear gradient lift force increases, the particles are pushed closer to the top and bottom of the channel walls, leading to a slightly stronger Dean drag force through the inner wall. Thus, particles preserve their previous lateral position. When the particles move further towards position 3 (the transition region), the velocity maxima are formed at the centre of the channel, resulting in a lift coefficient magnitude of zero and hence an absence of the lift force. However, because of the sudden directional curvature change (as the inner wall becomes the outer wall and vice versa), the curvature effect becomes negligible. Thus, the Dean drag force becomes insignificant. At that point, the particles follow the streamlines and preserve the previous lateral position until they reach the redistributed velocity profile region. Beyond position 3 (transition region), the direction of the curvature changes and it should be noted that whenever the curvature changes, the direction of the forces changes to the opposite direction. In position 4, because of the change in the curvature, the velocity maxima that are concentrated in the centre of the channel now shift towards W_2_. Again, the horizontal component of shear gradient lift force is through the outer wall, and the Dean drag force is the counterbalancing force at the centreline. In position 5, the velocity maxima draw closer to the inner wall, causing the shear gradient lift force on the particles to slightly decrease in both vertical and horizontal directions. This decrease leads to a shift in the particles’ vertical direction towards the zero Dean velocity line, and so the Dean drag force becomes weaker.

In case 2 (the transverse motion of medium particles) (Fig. 5c), while Dean drag force is in the opposite direction compared to case 1, horizontal component of shear gradient lift force direction is the same as that in situation 1. It is well known that an increase in flow velocity causes a decrease in the lift coefficient^55^. Hence, not only horizontal, but also vertical components of the lift force decrease with increasing flow velocity compared to case 1. As a result, the particles approach the vertical centre of the channel or beyond the zero Dean velocity line. Upon crossing this vertical position, the particles experience a change in the direction of the Dean drag force, which is now directed towards the outer wall.

In this case (Fig. 5c), when the medium particles are in position 1, where the velocity maxima are in the inner half of the channel, a combined effect of the Dean drag force and horizontal component of the shear gradient lift force (both of which are in the same direction) causes the particles to migrate through the outer wall. In this position, a slight defocusing trend can be seen in Fig. 3b(iv). The reason for this defocusing trend is the absence of the counter force near the centreline of the channel for medium particles. When the particles move through position 2, the velocity maxima approach the channel centre, causing a slight increase in the shear gradient lift force on the particles. The particles move closer to W_2_ under the influence of the shear gradient and Dean drag forces. When the particles move further towards position 3 (the transition region), the velocity maxima form at the centreline. Again, because of the sudden directional curvature change, the curvature effect become negligible so that the Dean drag force becomes insignificant. Now, the shear gradient lift force is the major force and pushes particles through the wall, whereas the wall-induced lift force is the balancing force. Beyond this position, the sudden change in outer and inner wall causes the Dean vortices to change direction, and the particles move through the higher shear area. In position 4, because of the curvature changes, the velocity maxima form in the inner half of the channel. The Dean drag force is through the outer wall. Therefore, the particles are pushed to the centreline from the wall under the influence of the shear gradient lift force and Dean drag force. Again, there is no balancing force, so that a slight defocusing trend can be observed near the outlet region, Fig. 3d(iv). In position 5, the velocity maxima draw closer to the centreline, causing the shear gradient lift force to push particles towards the outer wall, whereas the Dean drag force is through outer wall. An increase in defocusing behaviour can be noticed as particles approach to this position, Fig. 3d(iv).

This defocusing behaviour is not observed for large particles in case 3 (Fig. S2) due to scaling of the shear gradient lift force with 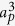. Thus, the magnitude of the vertical component of this force is still sufficient to push large particles above the zero Dean velocity line as in case 1, while it is not sufficient for medium particles.

An increase in Re_*C*_ results in a greater lift force compared to dean drag force (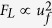, 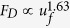). As a result, large particles migrate to W_2_. Force balance in this case is similar to case 1, except the transition region. Although velocity maxima are near the centreline in transition region for all cases, the shear gradient lift force becomes negligible in case 1 (particles are near the centreline) because of the difference in particle lateral positions, while particles experience a shear gradient lift force through the W_2_ in case 3 (particles are near the W_2_) in the transition region. As the particles approach the outer wall, the wall-induced lift force becomes more dominant and balances the other two forces (the Dean drag force and shear gradient lift force). The only countering force here is the wall-induced lift force near the channel wall. A slight lateral position difference between 15 and 20 μm particles can be observed when the Reynolds number is 38 such that the medium particles are slightly closer to the outer wall compared with the larger particles.

Without a full assessment of all three-dimensional effects, this result could at first glance be unexpected because the shear gradient and Dean drag forces change with 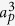 and *a*, respectively, so that the expected lateral position would be closer to the centreline for medium particles compared with large particles because the order of magnitude of the shear gradient results in a significant effect on large particles. However, the shear gradient also scales with 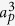 in the vertical direction and pushes larger particles to the top and bottom channel walls, where the Dean drag force is more dominant. Medium particles should be closer to the zero Dean velocity region. Therefore, the Dean drag force on large particles is greater than the Dean drag force on medium particles, so that the stable equilibrium position of large particles is observed to be closer to the centreline.

The main focus of this study is to investigate the effect of velocity maxima shift on the net inertial lift force (*F*_*L*_) and direction change of the dean Drag force (*F*_*D*_) according to the 3D position of particles in the microchannel. The change in the main flow velocity profile can directly affect *F*_*L*_ and *F*_*D*_, hence the balancing force and particle dynamics. Our simulation results do not consider the interaction between the particle and the flow. In fact, recent simulation studies show that flow field could be changed with the presence of the particles^58^,^60^. However, our simplified hypothesis can give a qualitative insight into the inertial microfluidics physics in curvilinear channels. On the other hand, running the simulations with particles for getting quantitative data is a challenging task due to certain reasons (code complexity, computational resources etc.), and it is our long-term vision to develop simulation methods, which are capable of performing such simulations.

Our findings suggest that carefully tailoring geometrical parameters could result in a curvilinear design, which could easily meet the requirement for a high throughput size based on separations for a vast variety of biomedical and other applications.

## Methods

### COMSOL Modelling

The COMSOL Multiphysics 4.2 software program (COMSOL Inc., Burlington, MA) was utilized to analyse fluid flow at multiple cross sections of the curved microchannels. The dimensions of the 3D model were determined based on the microchannel dimensions used in the experimental study shown in Fig. 1. A 3D Cartesian coordinate system (x, y, z) with the origin located at the cross sectional centre of the microchannel structure inlet was used. Full Navier–Stokes equations were solved using single phase and incompressible flow assumptions via the laminar flow model. For an incompressible and steady laminar flow, the governing Navier–Stokes equations can be expressed as


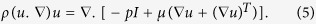


Various inlet velocities corresponding to Reynolds numbers 1–100 were considered at the inlet of the microchannels. A no-slip velocity condition on the walls and zero pressure at the outlet were chosen as the boundary conditions. Consequently, from the steady-state solution of the laminar flow, maximum Dean flow speeds and axial flow speeds were deduced, and Dean vortices were obtained using the velocity fields at different cross sections. The data obtained from the simulations match with the experimental observations and bolster the experimental results.

### Device Fabrication

PDMS (polydimethylsiloxane) (Sylgard 184, Dow Corning) microchannel devices were fabricated using the standard soft lithography microfabrication technique. Single-side polished 3” silicon wafers were coated with SU-8 3050 photoresist (Microchem Corp.) by means of a spinner (Dorutek) and exposed to UV light via a Mask Aligner UV-Lithography device (Midas System Co., Ltd., MDA-60MS Mask Aligner 4”). The image reversal acetate masks used in the lithography process were produced by a printer with 10,000 DPI (by CAD/Art Services, Inc.). The unexposed area was then developed using SU-8 Developer (Microchem Corp.). PDMS prepolymer and curing agent (Sylgard 184 silicone elastomer kit, Dow Corning) were mixed at a 10:1 (weight:weight) ratio in a plastic weighing dish and cast over the silicon master contained in a glass petri dish. The prepolymer mixture in the master was placed in a vacuum oven (Sheldon Manufacturing, Inc.) and degassed at low pressure (76 mmTorr) for 1 h to remove air bubbles and then cured for 12 h at 75 °C. Following curing, solid PDMS was peeled off from the master and cut with a scalpel. Inlet and outlet holes were formed using a 2 mm biopsy puncher. As a pre-process of bonding, microscopic glass slides were cleaned with isopropanol alcohol and deionized (DI) water along with PDMS moulds and then dried with N_2_ gas. After cleaning, both the PDMS channels and glass slide were placed in an Oxygen Plasma Device (Harrick Plasma Cleaner) and activated for 60 s. Thereafter, the PDMS bond surface was immediately brought into contact with the glass slide to create the enclosed form of the channel and was baked for 15 min at 75 °C on a hotplate (Dorutek) to strengthen the bonds.

### Sample Preparation

Fluorescent-labelled polystyrene particles (10 μm, Invitrogen; 15 μm, Invitrogen; 20 μm, Phosphorex) with an initial particle concentration of 1% were diluted in deionized (DI) water to obtain a final concentration of less than 0.01 wt.% for the focusing experiments, and a mixture of 15 μm and 20 μm particles was diluted in DI water for the particle focusing experiment demonstration. Exceedingly low particle fraction ratios were preferred in this study to avoid particle–particle interactions. For each set of experiment, a new microchannel is used for avoiding clogging or settlements of particles.

### Device Characterization/Experimental Setup

For each experiment, particle suspensions were filled in a 60 mL plastic syringe and injected into the microfluidic device at flow rates ranging from 100 to 3000 μl/min using a syringe pump (Harvard Apparatus PHD 2000). The flow rate was increased by 100 μl/min every 45s. Each experiment was repeated at least three times. Connections between the microfluidics device and syringe/collection tubes were made using TYGON tubing (IDEX Corp., IL) (internal diameter: 250 μm, length: 150 mm) and the corresponding fittings (IDEX Corp., IL). The device was mounted on an inverted phase contrast microscope (Olympus IX72) equipped with a (12-bit) charge coupled with a device camera (Olympus DP 72) and mercury lamp (Olympus U-LH100HG). For the fluorescence imaging, videos and image sequences of the particle motion were captured for each flow rate, and the exposure time was set at a high value of 600 ms. Olympus software (Olympus VS120-S5) and ImageJH software were used for fluorescence imaging and analysis, respectively. The use of ImageJH software emerged from the need for a composite stack of singular discrete frames to conduct a better analysis. To measure the migration length of particles and the focusing line thickness, a plotting line profile module was used across the channel width in the outlet and transition positions. Each line scan was then implemented in Origin Software to obtain a representative column of pixels in the Re_*C*_ maps. An example for determination of the focusing position and focusing width from fluorescent intensity graph at Re_*C*_ = 144 is given in Fig. S1.

## Additional Information

**How to cite this article**: Özbey, A. *et al*. Inertial Focusing of Microparticles in Curvilinear Microchannels. *Sci. Rep.* **6**, 38809; doi: 10.1038/srep38809 (2016).

**Publisher's note:** Springer Nature remains neutral with regard to jurisdictional claims in published maps and institutional affiliations.

## Supplementary Material

Supplementary Information
