# Supplementary material for: Inertial Focusing of Microparticles in Curvilinear Microchannels
Source: Sci Rep. 2016 Dec 19;6:38809. doi: 10.1038/srep38809 (PMC5171716; doi:10.1038/srep38809)
Supplement: Supplementary Information [file srep38809-s1.doc]

**Supplementary Information for**

**Inertial Focusing of Microparticles in Curvilinear Microchannels**

Arzu Özbey^a^, Mehrdad Karimzadehkhouei^a^, Sarp Akgönül^a^, Devrim Gözüaçık^b,c^, and Ali Koşar^a,c,*^

^a^Faculty of Engineering and Natural Science, Mechatronics Engineering Program, Sabanci University, Tuzla, Istanbul, Turkey 34956

^b^Faculty of Engineering and Natural Science, Biological Sciences and Bioengineering Program, Sabanci University, Tuzla, Istanbul, Turkey 34956

^c^Center of Excellence for Functional Surfaces and Interfaces for Nano-Diagnostics (EFSUN), Sabanci University, Tuzla, Istanbul, Turkey 34956

^*^Corresponding author, e-mail: [*kosara@sabanciuniv.edu*](mailto:kosara@sabanciuniv.edu), Tel: +90-2164839621, Fax: +90-2164839550


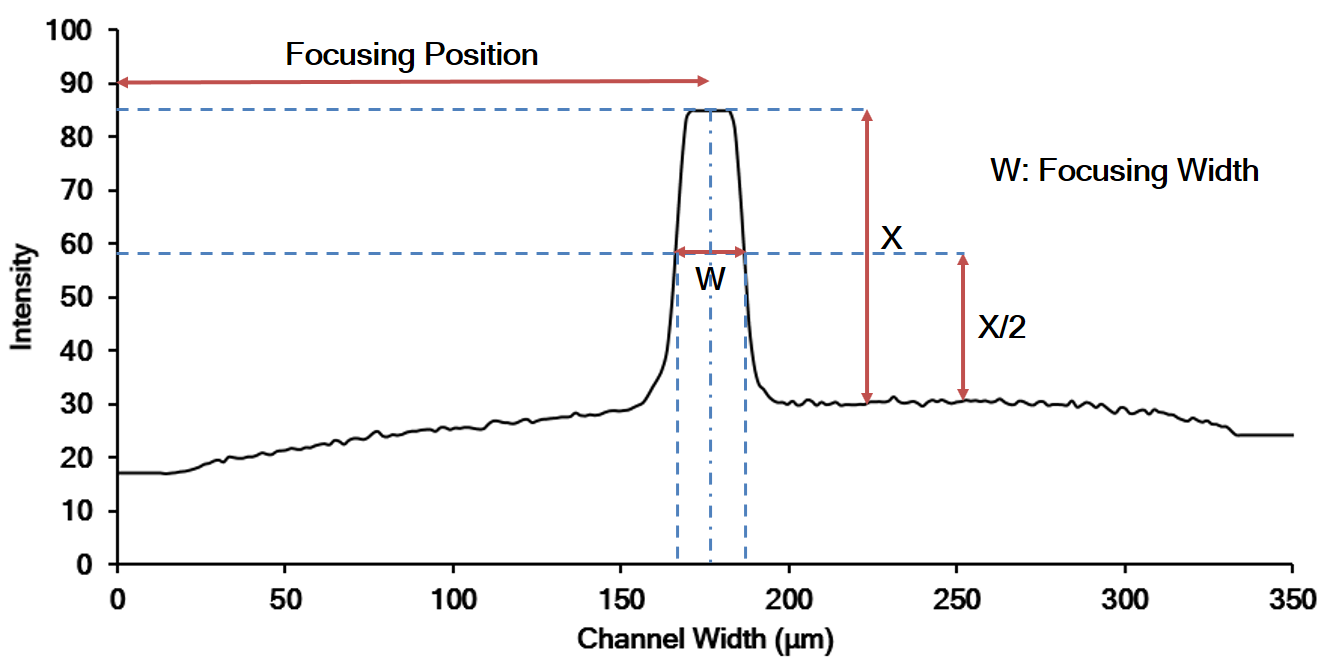


Fig. S1. Determination of the focusing position and focusing width from fluorescent intensity graph at $\mathrm{Re}_{C}$=144. (X: maximum intensity, W: Focusing width)

For each flow rate, separate videos from transition and outlet regions were captured and a set of 50 images was overlaid using the ImageJH software to quantify the exact focusing position and width. The plotting line profile module was used across the channel width in the outlet and transition positions to prepare the fluorescent intensity graph. Important parameters and a sample are shown in Fig. S1.


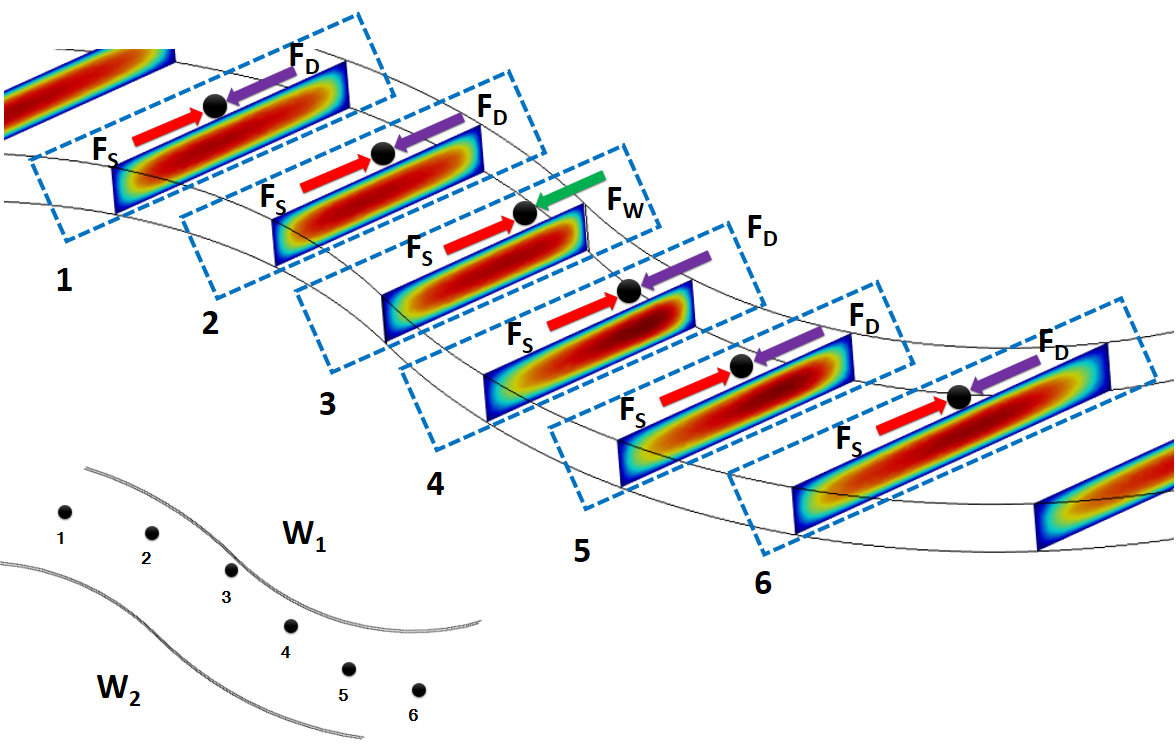


Fig. S2. Simulated velocity profile in the transition region and the forces acting on the particles where transverse motion of large particles is observed. Dominant forces acting on the particles are $F_{S}$ (shear gradient lift force), $F_{D}$ (Dean drag force) and $F_{W}$ (wall-induced lift force).
